# Molecular Fingerprint of Amphioxus Frontal Eye Illuminates the Evolution of Homologous Cell Types in the Chordate Retina

**DOI:** 10.3389/fcell.2020.00705

**Published:** 2020-08-04

**Authors:** Jiri Pergner, Anna Vavrova, Iryna Kozmikova, Zbynek Kozmik

**Affiliations:** Department of Transcriptional Regulation, Institute of Molecular Genetics of the Czech Academy of Sciences, Prague, Czechia

**Keywords:** chordates, eye evolution, light detection, vision, gene expression, photoreceptors, interneurons, Notch signaling

## Abstract

The evolution of the vertebrate eye remains so far unresolved. Amphioxus frontal eye pigment cells and photoreceptors were proposed to be homologous to vertebrate photoreceptors and retinal pigmented epithelium, based on ultrastructural morphology and gene expression analysis in *B. floridae*. Here, we present comparative molecular data using two additional amphioxus species, a closely related *B. lanceolatum*, and the most divergent *A. lucayanum*. Taking advantage of a unique set of specific antibodies we characterized photoreceptors and putative interneurons of the frontal eye and investigated its neuronal circuitry. Our results corroborate generally conserved molecular fingerprint among cephalochordate species. Furthermore, we performed pharmacological perturbations and found that the Notch signaling pathway, a key regulator of retina development in vertebrates, is required for correct ratios among frontal eye cell types. In summary, our study provides a valuable insight into cell-type relationships in chordate visual organs and strengthens the previously proposed homology between amphioxus frontal eye and vertebrate eyes.

## Introduction

The evolution of the vertebrate eye remains enigmatic (Lamb et al., 2007; Lamb, 2013). This is in particular due to the limited number of extant species at the invertebrate/vertebrate lineage transition (at the base of the chordate lineage). One such notable example is represented by cephalochordates (also known as amphioxus or lancelets), the most basally branching chordate clade. This clade includes three genera – *Branchiostoma*, *Asymmetron* and *Epigonichthys* and in total about 30 different species (Poss and Boschung, 1996). To date, the genomes of the three species of *Branchiostoma* have been sequenced (Holland et al., 2008; Putnam et al., 2008; Huang et al., 2014; Marletaz et al., 2018). Their analysis confirmed that cephalochordates did not undergo the two rounds of whole-genome duplication that occurred in vertebrates (Holland et al., 2008; Putnam et al., 2008; Huang et al., 2014; Marletaz et al., 2018). The phylogenetic relationship within the extant amphioxus lineage was investigated providing divergence time estimates. Molecular dating analysis based on whole-genome nuclear transcriptome revealed a divergence time of ∼120 Ma for *Asymmetron lucayanum* and *Branchiostoma floridae* (Yue et al., 2014), being comparable with that of the marsupial/placental split (Benton et al., 2009). This is somewhat less than ∼162 Ma estimated from mitochondrial gene sequences (Nohara et al., 2005; Kon et al., 2007). In either case, informative evolutionary divergence applies to *Branchiostoma* and *Asymmetron* pair (Yue et al., 2016). Much more recent diversification was found within the *Branchiostoma* genus (Igawa et al., 2017).

Comparison of differences and similarities between cephalochordates and vertebrates provides valuable information about ancestral chordate traits as well as vertebrate-specific innovations. Cephalochordate body plan possesses typical chordate characters like segmented muscles, dorsal hollow neural tube, notochord, perforated pharynx with gill slits and through gut - traits that can be found also in vertebrates. On the other hand, cephalochordates lack several vertebrate-specific characters, e.g., neural crest cells, paired appendages (fins or limbs), or well-developed paired sensory organs – eyes and ears (reviewed in Bertrand and Escriva, 2011). Amphioxus possesses four distinct photoreceptive organs: frontal eye, lamellar body, Joseph cells and dorsal ocelli (reviewed in Pergner and Kozmik, 2017). The frontal eye is considered as homolog of vertebrate eye based on its topology, ultrastructural morphology, gene expression pattern and circuitry (Lacalli et al., 1994; Lacalli, 1996; Vopalensky et al., 2012; Suzuki et al., 2015; reviewed in Pergner and Kozmik, 2017). The lamellar body is putative homolog of vertebrate pineal gland, mainly on the basis of its ultrastructural morphology, circuitry and topology (Ruiz and Anadon, 1991; Lacalli et al., 1994; Zieger et al., 2017). Possible vertebrate counterparts of Joseph cells and dorsal ocelli (both formed by rhabdomeric photoreceptors) are still matter of debate (reviewed in Pergner and Kozmik, 2017). The frontal eye is a single simple organ located at the tip of amphioxus cerebral vesicle – homolog of the vertebrate brain. Due to its simple organization, the frontal eye does not possess image-forming capacity. It is formed by only about six photoreceptor cells located in one horseshoe-shaped transversal row (Lacalli et al., 1994; Lacalli, 1996; Vopalensky et al., 2012). These cells were also designated as Row1 cells. Ultrastructural characterization of frontal eye demonstrated that Row1 photoreceptors are of ciliary morphology as are rods and cones (photoreceptors) in vertebrate retina (Lacalli et al., 1994). Row1 photoreceptors ultrastructure is, however, less elaborate than that of vertebrate photoreceptors (Lacalli et al., 1994). Anteriorly from Row1 photoreceptors lie nine pigment cells, arranged in three rows each consisting of three pigment cells (Lacalli et al., 1994). Posteriorly from photoreceptors lie three rows of putative homologs of vertebrate retinal interneurons and/or possibly also retinal ganglion cells (RGCs). These are the so-called Row2, Row3 and Row4 cells, respectively (Lacalli et al., 1994). The arrangement of these rows of cells is less organized than that of Row1 putative photoreceptors (Lacalli et al., 1994). The proposed homology between the frontal eye and vertebrate eyes was strongly supported by molecular studies in one cephalochordate species, *B. floridae*, showing that orthologous genes are expressed in both the frontal eye and vertebrate retina and pigmented epithelium (Vopalensky et al., 2012). Transcription factors Pax2/5/8, Mitf and Otx were detected in the developing row of frontal eye pigment cells (Vopalensky et al., 2012). Otx, Pax6, c-opsins and Gi subunit of trimeric G proteins were found to be expressed in frontal eye photoreceptors (Vopalensky et al., 2012). Taken together, these results were in concordance with gene expression profile of vertebrate photoreceptors and retinal pigmented epithelium (RPE) but provided limited information about possible homology between Row2, Row3, and Row4 cells of frontal eye and vertebrate retinal interneurons and/or RGCs. In vertebrates, Pax6 is expressed in most of the interneurons and RGCs and is required for their proper differentiation (Marquardt et al., 2001; Klimova and Kozmik, 2014). *B. floridae* Pax4/6 expression was not detected in Row2 cells but was instead found to be expressed in Row3 and Row4 cells (Vopalensky et al., 2012). Vopalensky et al. (2012) demonstrated that the Row2 neurons in *B. floridae* were positive for serotonin (alternatively called 5-hydroxytryptamine- further in the text marked as 5HT). More recently it was shown that some of the Row3 and Row4 neurons utilize glutamate as neurotransmitter (Pergner and Kozmik, 2017). Another criterion that can help to address homology between particular neuronal subtypes in the vertebrate retina and amphioxus frontal eye, is directionality of their axonal projections (used for example by Lacalli, 1996). Amacrine and horizontal cells form in vertebrate retina branches connecting neuronal layers laterally, while bipolar and ganglion cells form relay connections between retinal layers and visual processing center (Erskine and Herrera, 2014). Additionally, majority of RGCs in most vertebrates form contralateral projections (Erskine and Herrera, 2014). Based on electron microscopical studies and immunofluorecent imaging in *B. floridae* it was shown that Row2 cells form ipsilateral projections resembling bipolar cells (Lacalli, 1996; Vopalensky et al., 2012). Row3 cells form lateral contacts with each other resembling amacrine and horizontal cells, but also short irregular projections distantly resembling bipolar cells or RGCs (Lacalli, 1996). Some Row4 cells form contralateral projections resembling typical contralateral RGCs projections forming chiasma complex (Lacalli, 1996). Moreover, Lacalli (1996) observed that Row4 cells are in contact with Row2 cells. EM study also indicated that Row2 cell processes do not form any kind of terminals which questioned their ability to serve as interneurons (Lacalli, 1996). This could mean that they are involved in some kind of visual circuitry beginning with Row1 cells as photoreceptors, Row2 as interneurons, and Row4 as homologs of RGCs. Vopalensky et al. (2012) nevertheless observed that Row2 cell projections lead to an area of amphioxus cerebral vesicle that might be homologous to vertebrate visual processing center (tectum). The same was observed by Suzuki et al. (2015). A recent study focusing on homologies between amphioxus cerebral vesicle and particular regions of the vertebrate brain demonstrated that Row2 cell processes might indeed terminate in putative amphioxus proto-tectum of mesencephalic origin (Albuixech-Crespo et al., 2017) (reviewed in Pergner and Kozmik, 2017). It is, however, impossible to make any definitive conclusion about possible homology between Row3 and Row4 cells and vertebrate retinal interneurons and/or RGCs.

Limited data exist about the development of amphioxus frontal eye region and differentiation of neural progenitors into individual “retina” cell types. Expression of Otx and Pax4/6 genes whose orthologs are known for their crucial role in vertebrate eye development was detected in the early neurula stage in developing cerebral vesicle of *B. floridae* (Williams and Holland, 1996; Glardon et al., 1998). Both of these genes are nevertheless likely involved not only in frontal eye development but also in the development of other parts of amphioxus nervous system. It is of note, that differentiated c-opsins-positive photoreceptors were observed in *B. floridae* frontal eye not earlier than by the larval stage at 48 h post-fertilization (hpf) (Vopalensky et al., 2012). No information is currently available about the role of signaling cascades in the development of amphioxus frontal eye. Yet, conservation of gene regulatory networks orchestrated by specific signaling pathway may aid the general effort to assign homology between cell types across divergent species. In the present study we analyzed involvement of Notch signaling in the development of amphioxus photoreceptors and putative interneurons of the frontal eye. It is well established that Notch signaling regulates development of vertebrate photoreceptors. Inhibition of Notch signaling was shown to result in an increased numbers of photoreceptors in the mouse retina (Jadhav et al., 2006; Nelson et al., 2007). The previous molecular study of Vopalensky et al. (2012) was limited to a single amphioxus species, *B. floridae*, leaving uncertainty about general conservation of the observed gene expression fingerprint across all cephalochordates. In the present study we therefore provide comparative molecular data using three amphioxus species, *B. floridae*, *B. lanceolatum*, and the divergent *A. lucayanum*. Our results extend previous observations (Vopalensky et al., 2012) and provide novel insight into neural circuitry and development of the frontal eye. Combined, our study strengthens the previously proposed homology between amphioxus frontal eye and vertebrate eyes.

## Results

### General Conservation of Molecular Fingerprint Among Cephalochordates

**FIGURE 1:**
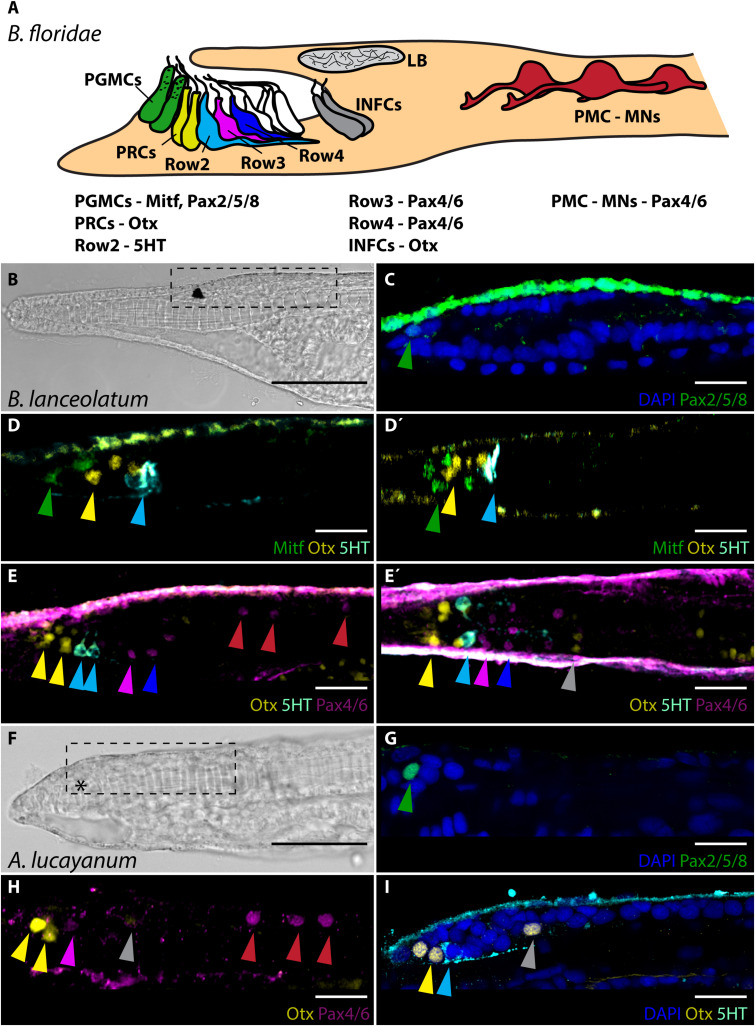
Molecular fingerprint of frontal eye in *B. floridae*, *B. lanceolatum* and *A. lucayanum*. **(A)** Schematic lateral view of molecular fingerprint of frontal eye cells in 48 hpf old *B. floridae* larva. Information about gene expressed in particular cells taken from study by Vopalensky et al. (2012). General morphology of frontal eye is same in *B. lanceolatum* and *A. lucayanum*. **(B)** Brightfield image of 4 dpf old *B. lanceolatum* larva. Dashed rectangle marks area of cerebral vesicle that is displayed in panels **(C–E’)** and also in Figures 2, 3, 4 and Supplementary Figures 2, 3. **(C)** Expression of Pax2/5/8. Green arrowhead highlights Pax2/5/8 expression in pigment cell. **(D)** Expression of Mitf, Otx, and 5HT. Green arrowhead marks expression of Mitf in pigment cell. Yellow arrowhead marks Otx signal in Row1 photoreceptors. Cyan arrowhead emphasizes 5HT expression in Row2 cells. **(D’)** Dorsal view on expression of Mitf, Otx, and 5HT shown in panel **(D)**. Arrowheads highlight same cells as in panel **(D)**. Gray arrowhead marks expression of Otx in infundibular cells. **(E)** Expression of Otx, 5HT, and Pax4/6. Yellow arrowhead marks Otx expression in Row1 photoreceptors. Cyan arrowhead points to 5HT expression in Row2 cells. Pink and blue arrowhead mark Pax4/6 expression in Row3 and Row4 cells, respectively. Red arrowheads highlight Pax4/6 expression in PMC neurons. **(E’)** Dorsal view on expression of Otx, 5HT, and Pax4/6 shown in panel **(E)**. Arrowheads mark same cells as in panel **(E)**. **(F)** Brightfield image of 8 dpf old *A. lucayanum* larva. Dashed rectangle marks area of cerebral vesicle that is displayed in panels **(G–I)**. Asterisks marks missing frontal eye pigmentation. **(G)** Expression of Pax2/5/8. Green arrowhead marks Pax2/5/8 expression in putative pigment cell. **(H)** Expression of Otx and Pax4/6. Yellow arrowheads points to Otx expression in Row1 photoreceptors. Pink arrowhead marks Pax4/6 expression in Row3 cells. Red arrowheads highlight Pax4/6 expression in PMC motoneurons. **(I)** Expression of Otx and 5HT. Yellow arrowheads point to Otx expression in Row1 photoreceptors. Cyan arrowhead marks 5HT expression in Row2 cells. Gray arrowhead marks Otx expression in infundibular cell. PGMCs, pigment cells; PRCs, photoreceptors; INFCs, infundibular cells; LB, lamellar body; PMC, primary motor center; MNs, motoneurons. Scale bar in panels **(B,F)** 50 μm. Scale bar in panels **(C–E’)** and **(G–I)** 20 μm.

Amphioxus undergo direct development comprising of severalmorhologically distinguishable stages defined in the past byHirakow and Kajita (1994). For the aim of this study the most important arefollowing stages (due to their connection with frontal eyedevelopment, which will be discussed later in the text): neurula N2 – hatching neurula with 3–5 somites, neurula N3 – with 9–10 somites, larval stage L1 – knife-shaped larva, the transition from embryo to larva, larval stage L2/3 – larva with open mouth and first gill slit open. First, we wanted to determine if the molecular fingerprint published previously for frontal eye of *B. floridae* 2-days old larvae (shown schematically in Figure 1A) (Vopalensky et al., 2012) is conserved across cephalochordates. To this end, we decided to characterize the molecular fingerprint of the frontal eye of closely related *B. lanceolatum*. The embryonic and larval development of *B. lanceolatum* appears significantly slower than that of *B. floridae* (Fuentes et al., 2007). For example, pigmentation of *B. lanceolatum* frontal eye appears not earlier than by 3 days post-fertilization (dpf) (Supplementary Figure 1) as compared to 1.5 dpf – 2 dpf in *B. floridae.* To perform gene expression analysis, we therefore opted to analyze developmentally matched larvae of the two species. As a consequence, we determined the molecular fingerprint of frontal eye in 4 days-old *B. lanceolatum* larva [1-gill slit larva with an already open mouth – L2/3 stage according to Hirakow and Kajita (1994)] (Figure 1B) and compared it in parallel experiments with fingerprint in 2 days-old *B. floridae* larva [2.5-gill slit larva – L3 stage according to Hirakow and Kajita (1994)]. Our analysis demonstrated that Pax2/5/8 (Figure 1C) and Mitf (Figures 1D,D’) was expressed in frontal eye pigment cells. Putative Row1 photoreceptors were Otx positive (Figures 1E,E’). *B. lanceolatum* Row2 cells utilize 5HT as neurotransmitter (Figures 1E,E’). Row3 and Row4 neurons were both shown to be Pax4/6 positive (Figures 1E,E’). We detected three pairs of Pax4/6 positive cells in primary motor center (PMC) (Figure 1E). Unfortunately, we were not able to detect c-opsins expression in putative photoreceptors of *B. lanceolatum* when using antibodies generated against *B. floridae* c-opsins (Supplementary Figure 2). This was probably due to low amino acid sequence conservation of opsins between *B. lanceolatum* and *B. floridae* [(Pantzartzi et al., 2018); discussed also in Bozzo et al. (2017)]. Taken together, molecular fingerprint of *B. lanceolatum* and *B. floridae* frontal eye is spatially conserved. The only observed difference appears to be in timing of frontal eye development.

Next, we characterized molecular fingerprint of frontal eye cells in *A. lucayanum*, representing the most divergent cephalochordate outgroup compared to the two *Branchiostoma* species. Despite close geographical localization of *B. floridae* and *A. lucayanum* and thus similar natural conditions of their habitat (Andrews, 1893), there is a significant difference in developmental timing between these two species (Holland and Holland, 1992, 2010; Holland and Yu, 2004). For unknown reasons *A. lucayanum* larvae survive for less than 2 weeks in laboratory conditions (Holland et al., 2015) but seem to stop developing already around 8 dpf (our observation). For our analysis we therefore selected 8 dpf-old *A. lucayanum* larvae, which were, however, still missing pigmentation of the frontal eye (Figure 1F). We detected Pax2/5/8 expression in putative pigment cell in the frontal eye of *A. lucayanum* (Figure 1G). *A. lucayanum* Otx was expressed in putative Row1 photoreceptors (Figure 1H). Row2 cells were found to be 5HT positive (Figure 1I). Pax4/6 was detected in putative Row3 and Row4 cells as well as three pairs of neurons in PMC (Figure 1H). As in the case of *B. lanceolatum* we were not able to detect c-opsins expression in *A. lucayanum* photoreceptors when using antibody generated against *B. floridae* c-opsins (Supplementary Figure 2).

### Expression of Lim Homeobox Transcription Factors in Row3 and Row4 Neurons

**FIGURE 2:**
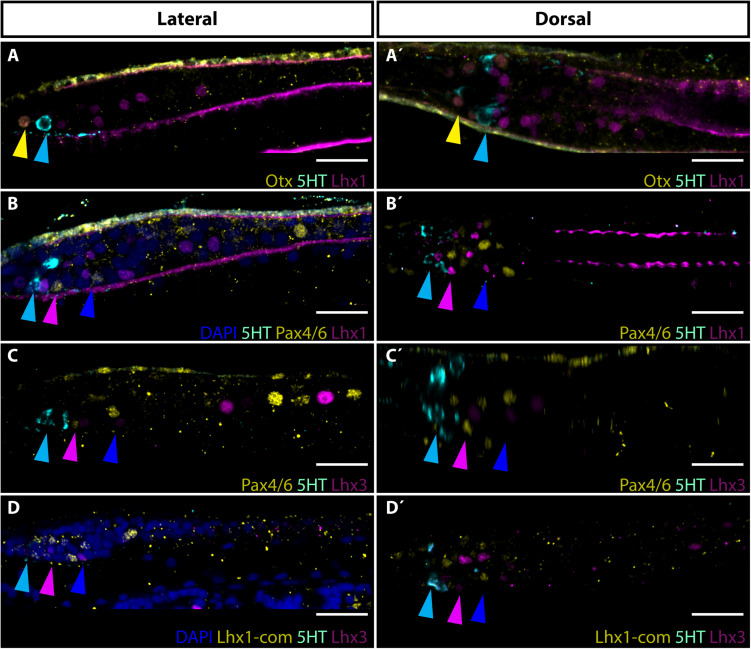
Expression of Lhx1 and Lhx3 in frontal eye neurons of 4 dpf old *B. lanceolatum* larva. **(A,A’)** Expression of Otx, 5HT and Lhx1. Yellow arrowhead marks co-expression of Otx and Lhx1 in Row1 photoreceptors. Cyan arrowhead hightlights 5HT expression in Row2 cells. **(B,B’)** Expression of 5HT, Pax4/6 and Lhx1. Cyan arrowhead marks 5HT expression in Row2 cells. Pink arrowhead highlights Pax4/6 and Lhx1 signal in Row3 cells. Blue arrowhead marks Pax4/6 and Lhx1 expression in Row4 cells. Noticeable is mutually exclusive expression of Pax4/6 and Lhx1 in Row3 and Row4 cells. **(C,C’)** Expression of Pax4/6, 5HT, and Lhx3. Cyan arrowhead marks 5HT expression in Row2 cells. Pink arrowhead points to Pax4/6 and Lhx3 signal in Row3 cells. Blue arrowhead marks Pax4/6 and Lhx3 expression in Row4 cells. Note, that Pax4/6 and Lhx3 are expressed in mutually exclusive manner in Row3 and Row4 cells. **(D,D’)** Expression of Lhx1 and Lhx3. Commercial antibody of rabbit origin was used for Lhx1 detection to allow co-staining with Lhx3. The commercial antibody provided same staining as in our lab developed amphioxus specific Lhx1 antibody (see Supplementary Figure 3). Cyan arrowhead marks 5HT expression in Row2 cells. Pink arrowhead indicates Lhx1 and Lhx3 expression in Row3 cells. Blue arrowhead points to expression of Lhx1 and Lhx3 in Row4 cells. Lhx1 and Lhx3 are expressed in distinct cellular populations. Scale bar 20 μm.

Proposed homology between Row3 and Row4 cells and vertebrate retinal interneurons was not adequately addressed in the initial molecular study (Vopalensky et al., 2012). To provide additional insight into evolutionary history of putative interneurons of the frontal eye we decided to determine the expression of Lim homeobox transcription factors Lhx1 and Lhx3 in amphioxus larvae. In vertebrates, Lhx1 and Lhx3 define distinct classes of retina interneurons. Lhx1 transcription factor was shown to be necessary for proper development of horizontal cells (Poche et al., 2007) whereas Lhx3 is expressed in bipolar cells (Elshatory et al., 2007). Although the expression of amphioxus Lhx1 and Lhx3 genes was previously investigated by RNA *in situ* hybridization (Wang et al., 2002; Langeland et al., 2006), no indication about possible presence of Lhx1 or Lhx3 in frontal eye region was obtained. Here, using specific anti-Lhx1 and anti-Lhx-3 antibodies, we were able to profile Lhx1 and Lhx3 genes at a single-cell resolution, and to demonstrate their expression in specific sets of frontal eye neurons. Lhx1 expression was found in both Row3 and Row4 cells of *B. lanceolatum* larvae (Figures 2B,B’). It is of note that Lhx1 was found in population of Row3/Row4 cells that was mutually exclusive to the Pax4/6-positive population of Row3/Row4 cells. Surprisingly, in addition to Row3 and Row4, Lhx1 immunoreactivity was also detected in Otx-positive photoreceptors (Figures 2A,A’). Lhx3 expression was likewise observed in Row3 and Row4 cells (Figures 2C,C’). As in the case of Lhx1 expression, Lhx3 expression appeared mutually exclusive to that of Pax4/6. To determine if Lhx1 and Lhx3 are co-expressed in Row3 and Row4 cells or if they are expressed in distinct subpopulation of these cells, we performed simultaneous detection of Lhx1 and Lhx3 using antibodies generated in different hosts. This was facilitated by our findings that a commercial antibody directed against Lhx1 of frog origin cross-reacts with amphioxus Lhx1. As shown in Supplementary Figure 3 anti-frog Lhx1 antibody labels the same cells as the antibody directed specifically against the amphioxus Lhx1. The signal obtained by using anti-frog Lhx1 antibody was weaker than when amphioxus Lhx1-specific antibody was used. We were nevertheless able to determine that Lhx1 positive and Lhx3 positive Row3 and Row4 cells populations are distinct from each other (Figures 2D,D’).

### Gene Expression Analysis of Proto-Tectum and Primary Motor Center

**FIGURE 3:**
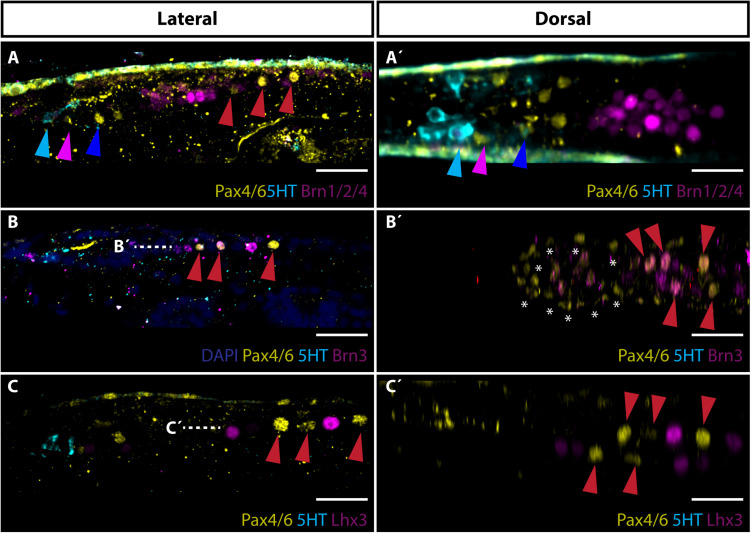
Expression of Brn1/2/4, Brn3 and Lhx3 with focus on visual processing center and primary motor center. **(A)** Lateral view of Brn1/2/4 expression. Brn1/2/4 is strongly expressed in neurons located posteriorly to Row4 (marked with blue arrowhead) and anteriorly from PMC cells (marked with red arrowheads). This area represents the putative amphioxus proto-tectum homologous to vertebrate tectum. Cyan arrowhead marks 5HT positive Row2 cells. Pink arrowhead indicates Pax4/6 positive Row3 cells. **(A’)** Dorsal view of staining presented in panel **(A)**. **(B)** Lateral view of Brn3 expression. Brn3 is co-expressed with Pax4/6 in two anterior pairs of Pax4/6 positive motoneurons localized in PMC. **(B’)** Dorsal view of staining showed in panel **(B)** at the level marked with dashed white line. Asterisks encircle strong background staining of cells at surface of the larvae. Evident is co-expression of Brn3 and Pax4/6 in PMC motoneurons marked with red arrowheads. **(C)** Lateral view of Lhx3 staining with stress on expression in motoneurons in PMC. Lhx3 positive neurons are localized exclusive to Pax4/6 PMC motoneurons (highlighted with red arrowheads). **(C’)** Dorsal view of Lhx3 staining shown in panel **(C)** at the level of white dashed line. Evident are Lhx3 and Pax4/6 positive populations of motoneurons in PMC. Scale bar 20 μm.

To gain more insights into the possible physiology of frontal eye we next focused on the development of proto-tectum (termed putative visual processing center in Suzuki et al., 2015) in amphioxus cerebral vesicle and PMC responsible for light-guided locomotor behavior using Brn1/2/4, Brn3, and Lhx3 as markers. The term proto-tectum is used here for the zone of amphioxus cerebral vesicle that might be homologous to precursor of both vertebrate pretectum and tectum, in the same meaning as was used in Lacalli (2018). In vertebrates, Brn2 (Pou3f2) and Brn4 (Pou3f4) were both detected in developing optic tectum (Brombin et al., 2011). In addition, Brn3 and Lhx3 were both shown to be important for development of vertebrate motoneurons (McEvilly et al., 1996; Thaler et al., 2002). We found that Brn1/2/4 was strongly expressed in the area of cerebral vesicle limited anteriorly by Row4 cells and posteriorly by Pax4/6 positive cells of PMC (Figures 3A,A’). This area represents the proto-tectum. Brn3 signal was detected in neurons of PMC (Figures 3B,B’). The first two anterior pairs of Pax4/6-positive PMC neurons co-expressed Brn3. Finally, Lhx3 was found to be expressed in PMC neurons in a mutually exclusive manner to Pax4/6 (Figures 3C,C’).

### Neurotransmitters in Frontal Eye Photoreceptors and Putative Interneurons

**FIGURE 4:**
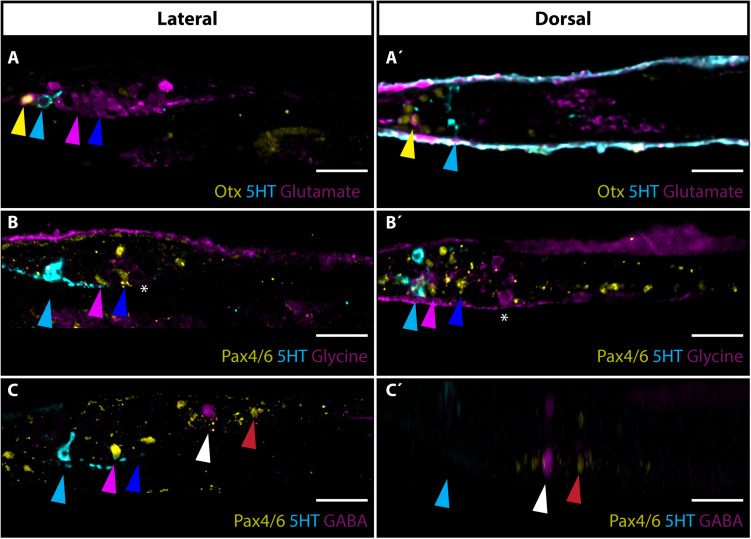
Neurotransmitters in amphioxus frontal eye neurons. **(A)** Expression of glutamate, Otx and 5HT in 4 dpf old *B. lanceolatum* larvae. Glutamate is utilized as neurotransmitters in Otx positive Row1 photoreceptors (marked with yellow arrowhead). Glutamate is used as neurotransmitter in most cells posterior to Row2 cells, including Row3 and Row4 cells (marked with pink or blue arrowhead, respectively). **(A’)** Dorsal view of staining shown in panel **(A)**. Noticeable is utilization of glutamate in Row1 photoreceptors. **(B)** Expression of glycine, Pax4/6 and 5HT in 4 dpf old *B. lanceolatum* larvae. Glycine is utilized as neurotransmitter by cells adjacent posteriorly to Row4 cells (marked by asterisks). **(B’)** Dorsal view of panel **(B)**. **(C)** Expression of GABA, Pax4/6, and 5HT in 4 dpf old *B. lanceolatum* larvae. GABA is not expressed in frontal eye neurons. Pair of GABA positive neurons is localized in Primary motor centre (PMC) anteriorly from large Pax4/6 positive neurons. **(C’)** Dorsal view of staining shown in panel **(C)**. GABA positive neurons are localized in Primary motor centre (PMC) anteriorly from large Pax4/6 positive neurons. Scale bar 20 μm.

Next, we focused on the utilization of neurotransmitters by frontal eye photoreceptors and putative interneurons. Glutamate was previously shown to be used as a neurotransmitter in *B. lanceolatum* photoreceptors (Pergner and Kozmik, 2017). In addition to the strong signal of glutamate found in Row1 photoreceptors (Figures 4A,A’), we observed that many (possibly all) Row3 and Row4 cells are glutamate-positive (Figures 4A,A’). We were able to detect glutamate in photoreceptors of distantly related *A. lucayanum* (Supplementary Figure 4). In *B. lanceolatum*, several cells posterior to Row4 cells were positive for glycine (Figures 4B,B’). We did not observe GABA in the frontal eye region (Figures 4C,C’). GABA positive neurons were only detected in one pair of PMC neurons located anteriorly from Pax4/6 positive neurons (Figures 4C,C’).

### Notch Signaling Inhibits Development of Frontal Eye Photoreceptors and Putative Interneurons

**FIGURE 5:**
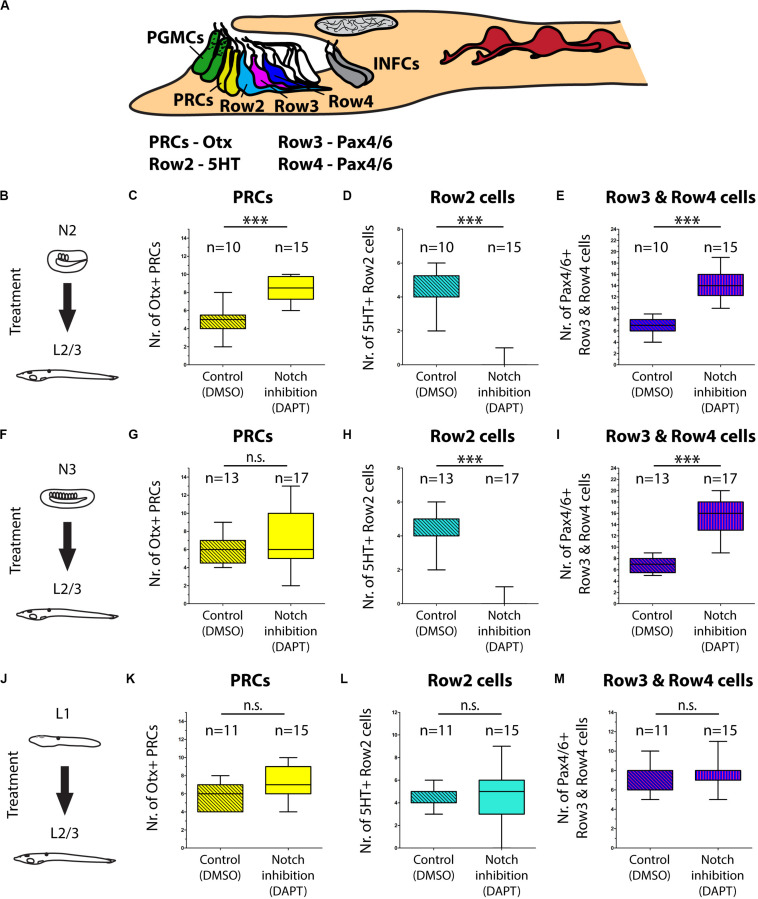
Effect of Notch inhibition on development of frontal eye in 4 dpf larvae of *B. lanceolatum*. **(A)** Scheme of cerebral vesicle of *B. lanceolatum* 4 dpf old larvae with highlighted landmarks and genes used for their identification. PGMCs, pigment cells; PRCs, photoreceptors; Row2, Row2 cells; Row3, Row3 cells; Row4, Row4 cells; INFCs, infundibular cells. **(B)** Scheme of experimental design for N2-L2/3 treatment. **(C)** Difference in numbers of photoreceptors (PRCs) after Notch inhibition (DAPT treatment). Significant increase in PRCs number after Notch inhibition was observed. **(D)** Difference in numbers of Row2 cells after Notch inhibition (DAPT treatment). Row2 cells did not develop in most embryos after Notch inhibition. **(E)** Difference in numbers of Row3 and Row4 cells after Notch inhibition (DAPT treatment). Inhibition of Notch led to Increase in number of Row3 and Row4 cells. **(F)** Scheme of experimental design for N3-L2/3 treatment. **(G)** Difference in numbers of photoreceptors (PRCs) after Notch inhibition (DAPT treatment). No significant change in PRCs number after Notch inhibition was observed. **(H)** Difference in numbers of Row2 cells after Notch inhibition (DAPT treatment). Row2 cells did not develop in most embryos after Notch inhibition. **(I)** Difference in numbers of Row3 and Row4 cells after Notch inhibition (DAPT treatment). Inhibition of Notch led to Increase in number of Row3 and Row4 cells. **(J)** Scheme of experimental design for L1-L2/3 treatment. **(K)** Difference in numbers of photoreceptors (PRCs) after Notch inhibition (DAPT treatment). No significant change in PRCs number after Notch inhibition was observed. **(L)** Difference in numbers of Row2 cells after Notch inhibition (DAPT treatment). No significant change of Row2 cells after Notch inhibition was observed. **(M)** Difference in numbers of Row3 and Row4 cells after Notch inhibition (DAPT treatment). No significant change of Row3 and Row4 cells after Notch inhibition was observed. Mann-Whitney two tailed test was used for calculation of statistical significance. Standard deviations are displayed.

**FIGURE 6:**
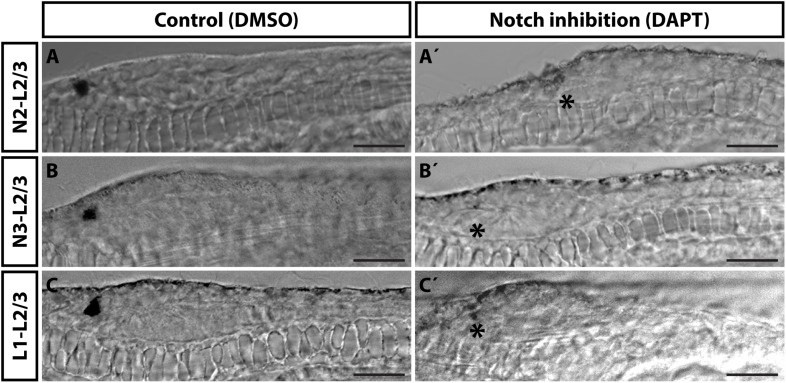
Effect of Notch inhibition on development of frontal eye pigmentation in 4 dpf *B. lanceolatum* larvae. Effect of Notch inhibition by DAPT on frontal eye pigmentation. **(A–C)** Control embryos treated with DMSO. **(A’–C’)** Embryos treated with 50 μM DAPT from N2 **(A’)**, N3 **(B’)** or L1 **(C’)** stage to L2/3 stage respectively. Treatment from L1 to L2/3 has lowest effect on frontal eye pigmentation – small remnants of pigment are visible. Asterisks mark missing pigmentation in DAPT treated larvae. Representative larvae are displayed. Scale bar 20 μm.

An evolutionarily conserved role of a signaling pathway may help to support homologies of cells in divergent species. In the present study we analyzed involvement of Notch signaling in the development of amphioxus photoreceptors and putative interneurons of the frontal eye. To this end, we used a pharmacological inhibitor of Notch signaling – DAPT (a gamma-secretase inhibitor) which blocks proteolytic cleavage of Notch intracellular domain (NICD). In the presence of DAPT the translocation of NICD to the nucleus and its normal function as a downstream effector of Notch signaling becomes abrogated. We treated *B. lanceolatum* embryos with DAPT starting from N2, N3, or L1 stage and determined the effect at larval stage L2/3 by marker analysis (experimental layout shown schematically in Figures 5B,F,J). More specifically, we counted Otx-positive photoreceptors, 5HT-positive Row2 cells and Pax4/6-positive Row3 and Row4 cells (markers of specific cell populations shown in the scheme in Figure 5A). Notch inhibition from N2 to L2/3 resulted in a significant increase in Otx-positive photoreceptor cells (Figure 5C and Supplementary Figure 5) and the absence of 5HT-positive Row2 cells (Figure 5D). Region of cerebral vesicle posterior to Otx-positive photoreceptors appeared morphologically altered following DAPT treatment precluding to distinguish Row3 and Row4 cell populations. Nonetheless, Notch inhibition led to a highly significant increase in Pax4/6-positive Row3/Row4 cells (Figure 5E and Supplementary Figure 5). It is of note that we did not observe a significant change in the number of putative infundibular Otx-positive cells (Supplementary Figure 6). DAPT treatment performed from N3 to L2/3 did not significantly influence the number of Otx-positive photoreceptors (Figure 5G) but resulted in the absence of 5HT-positive Row2 cells (Figure 5H), and in an increased number of Pax4/6-positive Row3/Row4 cells (Figure 5I). Finally, DAPT treatment from L1 to L2/3 stage did not affect any of the investigated cell types – neither the frontal eye photoreceptors nor putative interneurons (Figures 5K–M). Inhibition of Notch signaling in all treatment regimens led to the absence of frontal eye pigmentation, with the lowest effect being visible for treatment from L1 to L2/3 stage (Figure 6).

## Discussion

**FIGURE 7:**
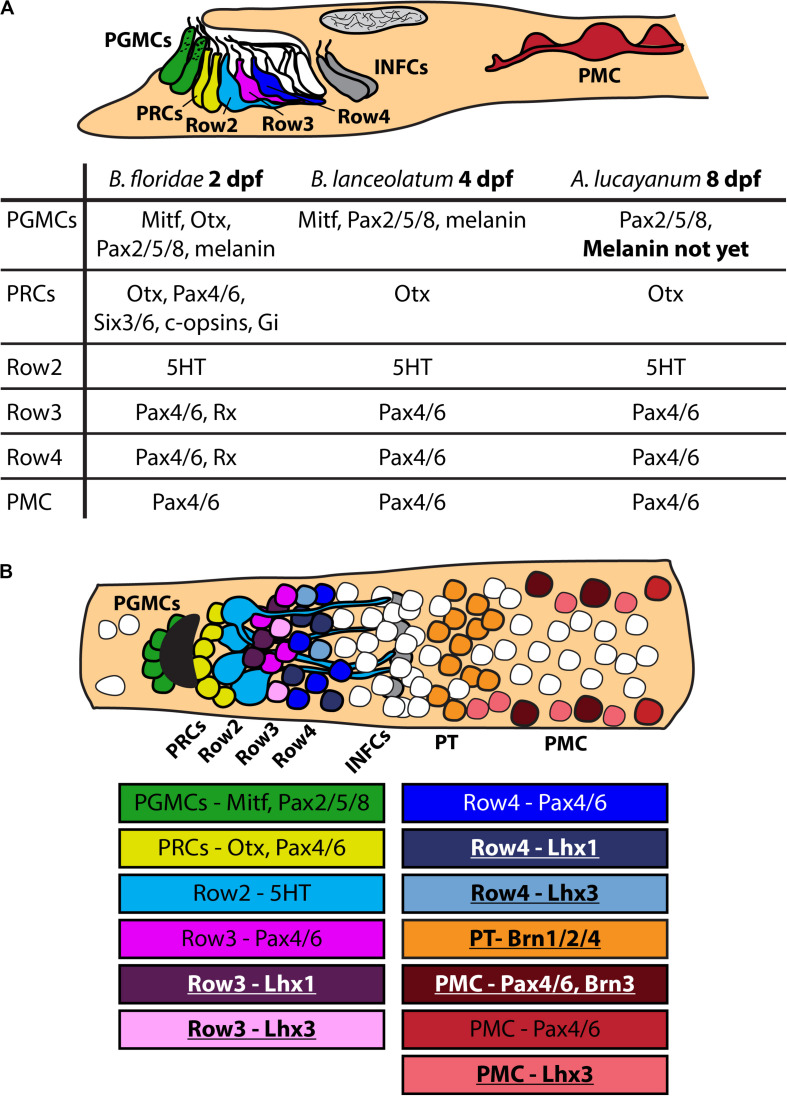
Scheme of molecular fingerprint of amphioxus frontal eye. **(A)** Schematic summary of molecular fingerprint of frontal eye in *B. floridae*, *B. lanceolatum* and *A. lucayanum*. Data for *B. floridae* were taken from Vopalensky et al. (2012). Data about molecular fingerprint in *B. lanceolatum* and *A. lucayanum* come from this study. Differences between particular species are emphasized using bold letters – noticeable is difference in frontal eye developmental timing and absence of frontal eye pigmentation in *A. lucayanum*. It was impossible to detect some antigens in *B. lanceolatum* and *A. lucayanum* using antibodies raised against *B. floridae* antigens (e.g., c-opsins). **(B)** Scheme of dorsal view on amphioxus cerebral vesicle with focus on frontal eye cells. Only some genes characteristic for particular cellular populations are listed. Underlined names of genes highlight data about gene expression coming from this study. PGMCs, pigment cells; PRCs, photoreceptors; Row2, Row2 cells; Row3, Row3 cells; Row4, Row4 cells; PT, proto-tectum; PMC, primary motor center.

The first aim of our study was to determine, whether previously reported frontal eye molecular fingerprint in *B. floridae* (Vopalensky et al., 2012) is conserved among cephalochordate species. The two species we selected for analysis are represented by a closely related *B. lanceolatum*, and the most distantly related *A. lucayanum*. We found that the overall molecular fingerprint of frontal eye pigment cells, photoreceptors and putative interneurons (Row2, Row3, and Row4 cells) was conserved among the three cephalochordate species (summarized in Figure 7A). However, we observed several conspicuous differences. Heterochrony in the appearance of the frontal eye between *B. lanceolatum* and *B. floridae* corresponds reasonably well with the overall differences in developmental timing. These two species develop at different temperatures which leads to a generally faster development of *B. floridae* embryos growing in warmer water (summarized in Fuentes et al., 2007). Heterochrony in frontal eye development observed between *B. floridae* and *A. lucayanum* is unexpected since both species inhabit relatively close geographical localities and develop at similar temperatures. No melanin-based pigmentation of the frontal eye in *A. lucayanum* was detected even by 8 dpf while the same type of pigmentation in the 1st dorsal ocellus was readily observed (Supplementary Figure 7). Lack of cross-reactivity of antibodies directed against *B. floridae* ciliary opsins toward *A. lucayanum* c-Ops1 and c-Ops3 precluded detection of their expression in the frontal eye as possible evidence of its functionality. Nevertheless, our results showed that 5-HT-positive Row2 cells are present in 8 dpf-old *A. lucayanum* larva, and Otx-positive photoreceptors produce glutamate (Supplementary Figure 4) indicating that both photoreceptors and Row2 cells are terminally differentiated and physiologically active by this developmental stage. The absence of frontal eye pigmentation, however, challenged the potential role of frontal eye in light-guided behavior of *A. lucayanum*, when compared with previously published work on Florida amphioxus. The study of Stokes and Holland (1995) demonstrated that directional light source triggers *B. floridae* larvae to adopt a specific vertical orientation in the water column which allows photoreceptors to be shielded by the pigment cells of the frontal eye (Stokes and Holland, 1995). Although not shown experimentally, we speculate that due to lack of pigmentation 8 dpf-old *A. lucayanum* larva might be unable to orient suggesting that the frontal eye can only function as a non-directional photoreceptive organ at this stage of development.

**FIGURE 8:**
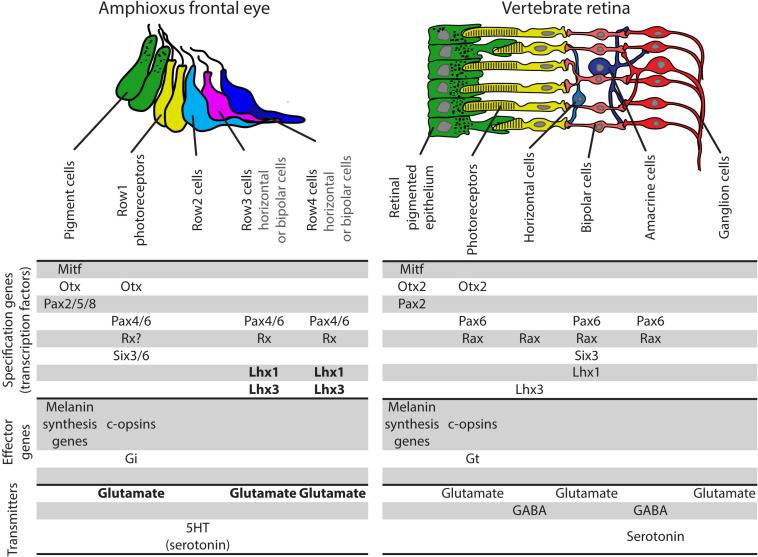
Comparison of gene expression pattern in amphioxus frontal eye and vertebrate retina and RPE. Homology between amphioxus frontal eye pigment cells and photoreceptors and vertebrate RPE and photoreceptors is strongly supported based on molecular fingerprint. Row2 cells of amphioxus frontal eye do not express typical marker of any subpopulation of vertebrate retinal interneurons. Row3 and Row4 frontal eye populations share same gene expression pattern. Interestingly, both of these populations express markers typical for two totally different populations of vertebrate retinal interneurons – bipolar or horizontal cells. See text for more information. Data for frontal eye and vertebrate eyes molecular fingerprints originally taken from Bassett and Wallace (2012), Kolb (2011), Swaroop et al. (2010), Vopalensky et al. (2012) and reviewed in Pergner and Kozmik (2017). Bold font marks data coming from this study. Gi, Gi alpha subunit of trimeric G proteins; Gt, transducing, Gt alpha subunit of trimeric G protein.

Previous studies of amphioxus frontal eye did not yield much information about putative interneurons Row3 and Row4 cells. So far, only Pax4/6 was found to be expressed in Row3 and Row4 cells. We chose to analyze expression of amphioxus Lhx1, Lhx3, and Brn3 due to their well-described function in vertebrate retina. Lhx1 was shown to be necessary for proper development of horizontal cells (Poche et al., 2007), Lhx3 is expressed in bipolar cells (Elshatory et al., 2007), and Brn3 is crucial for development of RGCs (Liu et al., 2000; Quina et al., 2005). We found that cells in Row3 and Row4 express both Lhx1 and Lhx3 (summarized in Figure 7B). In contrast, Brn3 expression was not detected in any of either Row3 or Row4 cells. Lack of Brn3 staining does not exclude the presence of putative homologs of RGCs in this area of the frontal eye but it makes it currently less likely. One cannot rule out that amphioxus homologs of RGCs are represented by dorsal ocelli, a scenario based primarily on dorsal ocelli morphology and the fact that they express rhabdomeric type opsin melanopsin (Koyanagi et al., 2005) (see also discussion in Pergner and Kozmik, 2017). Combined, our data suggest that cellular populations in Rows3/4 may be homologous to bipolar or horizontal cells (summarized in Figure 8).

To provide more insight into neuronal circuitry, we aimed to determine the nature of neurotransmitters utilized by individual frontal eye neurons. In vertebrates, most of the cells forming visual circuitry – photoreceptors, some populations of bipolar cells and most RGCs – utilize excitatory neurotransmitter glutamate (reviewed in Kolb, 2011). The same can be observed in amphioxus, where photoreceptors, Row3 and Row4 cells (putative interneurons) were found to be positive for glutamate (Figure 4). Some populations of amacrine cells in the vertebrate retina were found to be positive for inhibitory neurotransmitters glycine and GABA. Their proposed role is to modulate the transmission of the visual signal. We were unable to detect GABA or glycine in the region of putative frontal eye interneurons. Glycinergic neurons were instead found in the region where processes of Row2, Row3, and Row4 cells terminate. Here, glycinergic neurons might act as a neuromodulator of frontal eye circuitry. The presence of GABA was restricted to PMC motoneurons and might thus act in the modulation of motoneurons response to light stimuli. Our previous study revealed direct innervation and indicated the paracrine release of serotonin from Row2 cells in the tegmental neuropil in the posterior cerebral vesicle of *B. floridae*, reminiscent of retinohypothalamic projections in the vertebrates (Vopalensky et al., 2012). In the present study, we confirmed the restricted expression of 5-HT in Row2 cells using two other cephalochordate species, *B. lanceolatum*, and *A. lucayanum*, indicating a conserved nature of this molecular signature. The usefulness of serotonin-positivity in Row2 cells for drawing homologies between cell-types in the frontal eye and vertebrate retina nevertheless remains limited. 5-HT is produced by vertebrate photoreceptors, as a precursor for melatonin synthesis, and by amacrine cells, but the role of 5-HT as a neuromodulator in the vertebrate retina is not well understood (reviewed in Masson, 2019). Serotogenic neurons in the vertebrate brain are known to act as modulators of GABAergic and glutamatergic neurons (Ciranna, 2006). One can speculate that Row2 cells might act similarly as modulators of signal from photoreceptors to Row3 and Row4 cells. Row3 and Row4 cell axons terminate in the region of cerebral vesicle that likely serves as a visual processing center homologous to vertebrate optic tectum. In vertebrates, the expression of several POU transcription factors was observed in the developing tectum (Brombin et al., 2011). These included Brn2 (Pou3f2) and Brn4 (Pou3f4) genes, and a gene from Brn3 (Pou4f) family. Using specific antibodies we detected expression of Brn1/2/4, a single amphioxus ortholog of the vertebrate Brn2 and Brn4, in the putative tectum. The expression of amphioxus orthologs of Brn3 and Lhx3 was found in PMC motoneurons correlating well with the known role of Brn3 and Lhx3 in the development of vertebrate motoneurons (McEvilly et al., 1996; Thaler et al., 2002). Combined, the available data suggest that the frontal eye circuitry might be built upon similar logics as in vertebrates. We propose that amphioxus frontal eye circuitry starts with Otx positive photoreceptors utilizing excitatory neurotransmitter glutamate. Then it continues with Pax4/6 positive Row3 and/or Row4 cells (putative interneurons) utilizing also glutamate. Serotonin positive Row2 interneurons as well as glycine positive neurons posterior to Row4 cells might serve as inhibitory neurons modulating frontal eye response. Visual stimulus is then processed by neurons, which develop in Brn1/2/4 positive putative tectum. This would mimic the situation in vertebrates where photoreceptors and neurons transmitting the signal - bipolar and ganglion cells – utilize glutamate and the glycinergic amacrine cells serve as inhibitory modulators.

**FIGURE 9:**
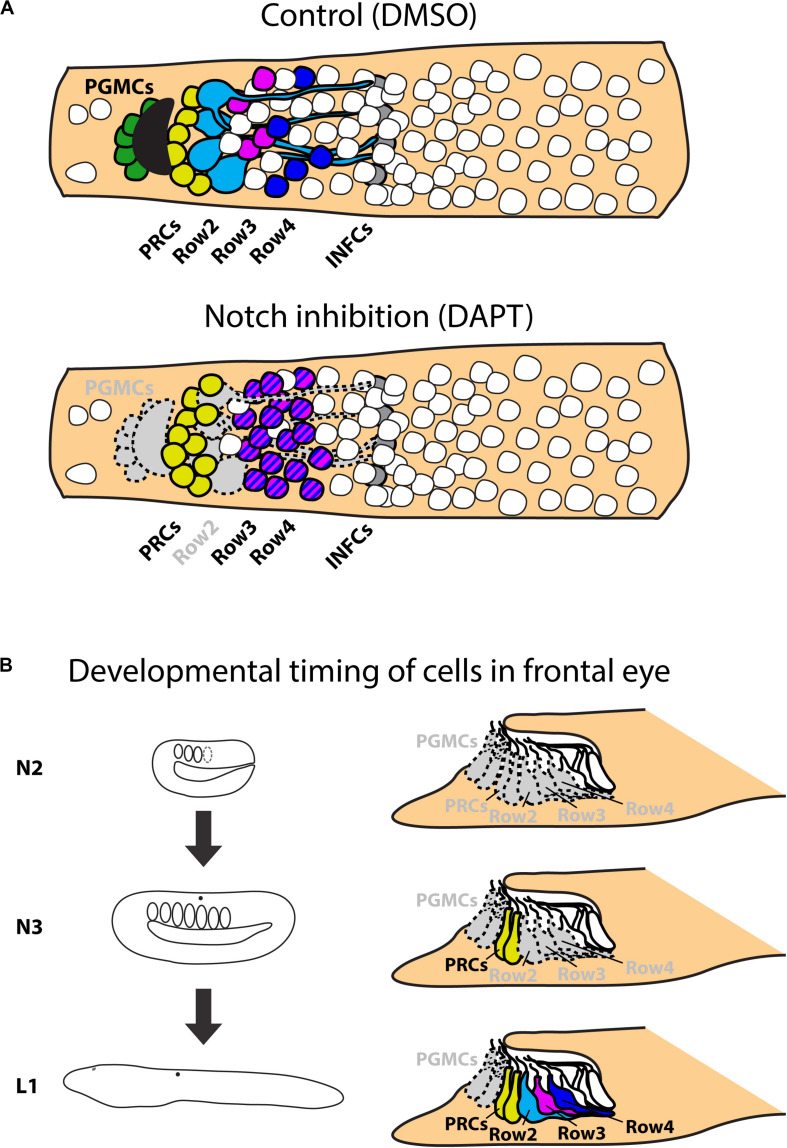
Scheme of results of Notch treatments. **(A)** Schematic representation of effect of Notch inhibition on frontal eye development. Inhibition of Notch signaling leads to absence of frontal eye pigmentation and serotonin positive Row2 neurons. Notch inhibition leads to increase of Otx positive photoreceptors and Pax4/6 positive Row3 and Row4 cells. Treatment does not influence Otx positive putative infundibular cells. **(B)** Scheme of effect of Notch inhibition on developmental timing on frontal eye neurons. Continuous treatments from N2 stage influence development of all monitored cell types – PGMCs; PRCs; Row2; Row3 and Row4 cells. Treatment from N3 stage does not have significant effect on development of PRCs, but affects all other cell types. Treatment starting from L1 affect only development of PGMCs. All other cell types develop normally. PGMCs, pigment cells; PRCs, photoreceptors (Row1 cells); Row2, Row2 cells; Row3, Row3 cells; Row4, Row4 cells; INFCs, infundibular cells.

To provide an insight into the gene regulatory mechanisms underlying frontal eye development and specification of its unique cell types we performed pharmacological treatments during amphioxus embryonic and larval development. We specifically focused on Notch signaling cascade, since in vertebrates the inhibition of Notch signaling leads to an increase in the number of photoreceptors (Jadhav et al., 2006). Our analysis has shown that Notch inhibition similarly results in an increased number of photoreceptors in the amphioxus frontal eye. In addition, following Notch inhibition we observed a highly significant increase in the number of the putative interneurons present within Row3 and Row4 (summarized in Figure 9A). In this context it is interesting to note that overexpression of Delta (ligand of Notch signaling) in vertebrates leads to a decrease of specific later-born retina interneurons (bipolar and amacrine cells) (Dorsky et al., 1997). In contrast, the number of early born neurons (ganglion or horizontal cells) was increased after Delta overexpression indicating that the phenotype was caused by changing the competence of retinal progenitor cells. Precise timing of pharmacological interventions allowed us to uncover windows of competence for differentiation of frontal eye cell types (summarized in Figure 9B). Notch inhibition at the earliest developmental stage (neurula stage N2) led to increased number of photoreceptors, Row3, and Row4 cells, and a complete absence of Row2 cells. The responsiveness of photoreceptor lineage to Notch signaling was lost by the neurula stage N3. In contrast, the fate of Row2, Row3, and Row4 could still be affected by DAPT treatment performed at N3 but not at larval stage L1. Finally, differentiation of melanin-producing pigment cells was sensitive to Notch inhibition applied in any of the examined stages. Differential time window sensitivity is likely related to the onset of differentiation of a particular cell type. Combined, Notch signaling is involved in the regulation of correct ratios of neuronal cell types within amphioxus frontal eye, i.e., the function that appears to be analogous to that described for the vertebrate retina.

## Materials and Methods

### Animal Culture and Embryos Fixation

*Branchiostoma lanceolatum* ripe adults were obtained from Argeles-sur-Mer (France) in April (before natural spawning season) and immediately transported to the Institute of Molecular Genetics of the Czech Academy of Sciences (Prague, Czechia). The animals were kept in a day/night cycle of 14/10 h. About 20–25 individuals were retained in 5 l of natural sea water, 17°C cold and fed with algae *Tisochrysis lutea* (CCMP463). Spawning was induced by temperature shift as described previously in Fuentes et al. (2007) during the natural spawning season period (May–July). Embryos were raised in 19°C to achieve normal development.

*Branchiostoma floridae* (Florida amphioxus) adults were collected in Old Tampa Bay, Florida during summer spawning season and transported to the Institute of Molecular Genetics of the Czech Academy of Sciences (Prague, Czechia). Individuals were kept in 5 l of natural sea water, 23°C cold and fed daily with algae. Before spawning, the individuals were shifted for at least 6 weeks to 17°C. Spawning was induced by temperature shift to 28°C for 30 h. Animals were separated into 0.5 l plastic cups with 30–50 ml of sea water 1 h before artificial sunset (switching off lights). Most of the animals spawned within 1 h after switching off lights. The spawning efficiency was about 20–80%. Embryos were raised in 25°C to achieve normal development. Embryos of both species were fixed with 4% PFA/MOPS [0.1 M 3-(N-morpholino)propanesulfonic acid, 2 mM MgSO4, 1 mM EGTA, 0.5M NaCl, pH 7.5] for 15 min on ice and transferred through series of 30, 70% methanol mixed with 1× PBS 0,1% Triton-X100 (PBT) to 100% methanol. Samples were stored in -20°C.

### Antibody Preparation

Amphioxus-specific antibodies were prepared as described in details earlier in Bozzo et al. (2017). Briefly, selected protein coding sequences were cloned into pET42a(+) vector (Novagen), sequenced and introduced into BL21 (DE3) RIPL bacteria (Stratagen). A total volume of 500 ml of fresh LB medium was inoculated with an overnight culture grown in presence of chloramphenicol (12.5 μg/ml) and kanamycin (30 μg/ml). Bacteria were grown at 37°C at 200 RPM until the culture reached OD600 0.6 and then induced by addition of 0.5 mM IPTG for 3 h. Cells were harvested at 6,000 × g for 10 min and the pellet stored at -80°C until further processing. The pellet was resuspended in Lysis buffer (6M guanidine hydrochloride, 0.1 NaH_2_PO_4_, 0.01 Tris-HCl, pH 8.0, supplemented with fresh beta-mercaptoethanol – final concentration 20 mM). The suspension was sonicated 6 × 20 s and incubated 3 h at room temperature (RT). Lysate was centrifuged at 10,000 × *g* for 10 min. Supernatant was transferred to new tube and incubated with Ni-NTA agarose beads (Qiagen) equilibrated with Urea buffer (8 M urea, 20 mM Tris. Cl, 50 mM NaH_2_PO_4_, 100 mM NaCl, pH 8.0, supplemented with fresh beta-mercaptoethanol to a final concentration of 20 mM). The suspension was incubated on rotating platform overnight (ON) at RT. On the next day, the beads were washed two times with 40 ml Urea buffer and loaded onto a chromatographic column (Bio-Rad). The column was washed with Urea buffer with decreasing pH (8.0–6.8). His-Tagged protein was eluted with Urea buffer pH 4.2 into several 1 ml aliquots. pH was immediately increased to 7.5 by addition of 1M Tris-HCl pH 8.0. Protein concentration was measure with Protein Assay Reagent (Bio-Rad). Three mice of the B10A-H2xBALB/CJ strain were immunized four times in 4 weeks’ intervals with 30 mg of purified protein in PBS mixed with Freund’s adjuvant (Sigma-Aldrich). An aliquot of serum was collected 10 days after the 3rd and 4th immunization. Serum following 4th immunization was mostly used for immunofluorescent staining.

### Immunofluorescent Staining

Embryos were transferred from 100% methanol to PBT through series of 70 and 30% methanol in PBT. Following three 15 min washes in PBT, the embryos were incubated for at least 1 h in blocking solution at RT. Next, the embryos were incubated ON at 4°C with primary antibodies. On the next day, the embryos were washed five times with PBT at RT (each wash at least 20 min). Subsequently, the samples were incubated with secondary antibodies and DAPI (1 mg/ml) for at least 3 h at RT. Embryos were then washed five times in PBT at RT and mounted on slides. Three layers of Scotch tape between the slide and the coverslip were used as spacer. The images were taken using Leica SP5 or SP8 confocal microscope and processed in FIJI image analysis software. The adjustment included use of filtering for improving signal/noise ratio and color balance adjustment. For final figures projection of several optical sections usually covering about 5 μm of embryo were used. Final figures were assembled in Adobe Photoshop CS4.

The following “home-made” primary antibodies directed against amphioxus proteins were used (dilution of antibody is indicated): mouse anti-Otx (1:500) (Vopalensky et al., 2012), rabbit anti-Otx (1:800) (Vopalensky et al., 2012), mouse anti-Pax4/6 (1:500), rabbit anti-Pax4/6 (1:500) (Vopalensky et al., 2012), mouse anti-Pax2/5/8 (1:500), mouse anti-Brn3 (1:500), mouse anti-Brn1/2/4 (1:500), mouse anti-Mitf (1:500), rabbit anti-Mitf (1:500) (Vopalensky et al., 2012), mouse anti-Lhx1 (1:500) (Bozzo et al., 2017), mouse anti-Lhx3 (1:500) (Bozzo et al., 2017), mouse anti-c-opsin1 (1:250) (Vopalensky et al., 2012), mouse anti c-opsin3 (1:250) (Vopalensky et al., 2012). The specificity of “home-made” antibodies against Lhx1, Lhx3, Brn3, and Brn1/2/4 was validated as previously described (Vopalensky et al., 2012) by Western blotting (Supplementary Figure 8) and by immunostaining with antibody pre-adsorbed with antigen (Supplementary Figure 9). The following commercially available primary antibodies were used: rabbit anti-Glutamate (Sigma-Aldrich, ref. nr. G6642) (1:250), rabbit anti-Glycine (kindly provided by Dr. Pow) (1:250), rabbit anti-GABA (Sigma-Aldrich, ref. nr. A2052) (1:250), goat anti-Serotonin (Abcam, ref. nr. ab66047) 1:1000, rabbit anti-Lhx1 (Chemicon, ref. nr. AB3200). Following secondary antibodies were used at dilution 1:500: donkey anti-mouse (ThermoFisher Scientific, ref. nr. A31571); donkey anti-goat (ThermoFisher Scientific, ref. nr. A21206); donkey anti-rabbit (ThermoFisher Scientific, ref. nr. A21432).

### Chemical Manipulations of Developing Embryos

All treatments were performed on *B. lanceolatum*. Embryos were raised in 19°C to achieve normal development. The embryos were treated from N2, N3 neurula stages and L1 to L2/3 larval stage based on staging by Hirakow and Kajita (1994). Embryos were separately treated with Notch inhibitor DAPT (Cayman chemical) in final concentration 100 μM and 10 μM im DMSO. The final concentration was max. 0.2%. Control embryos were raised in 0.2% DMSO in sea water.

Otx positive photoreceptors, 5HT positive Row2 cells and Pax4/6 positive Row3 and Row4 cells were counted in control and treated larvae in Z-stacks on confocal Leica TCS SP5 and Leica TCS SP8 microscope. Graphs and statistical analysis were performed in Graphpad software. Mann-Whitney two tailed test was used for calculation of statistical significance.

## Data Availability Statement

The raw data supporting the conclusions of this article will be made available by the authors, without undue reservation.

## Author Contributions

JP and ZK designed the study, conceived the experiments, and wrote the manuscript. JP, ZK, and AV performed the experiments. IK and ZK provided new reagents. All authors have read and approved the manuscript.

## Conflict of Interest

The authors declare that the research was conducted in the absence of any commercial or financial relationships that could be construed as a potential conflict of interest.
