# Supplementary material for: Molecular Fingerprint of Amphioxus Frontal Eye Illuminates the Evolution of Homologous Cell Types in the Chordate Retina
Source: Front Cell Dev Biol. 2020 Aug 4;8:705. doi: 10.3389/fcell.2020.00705 (PMC7417673; doi:10.3389/fcell.2020.00705)
Supplement: Supplementary file 1 [file Data_Sheet_1.PDF]

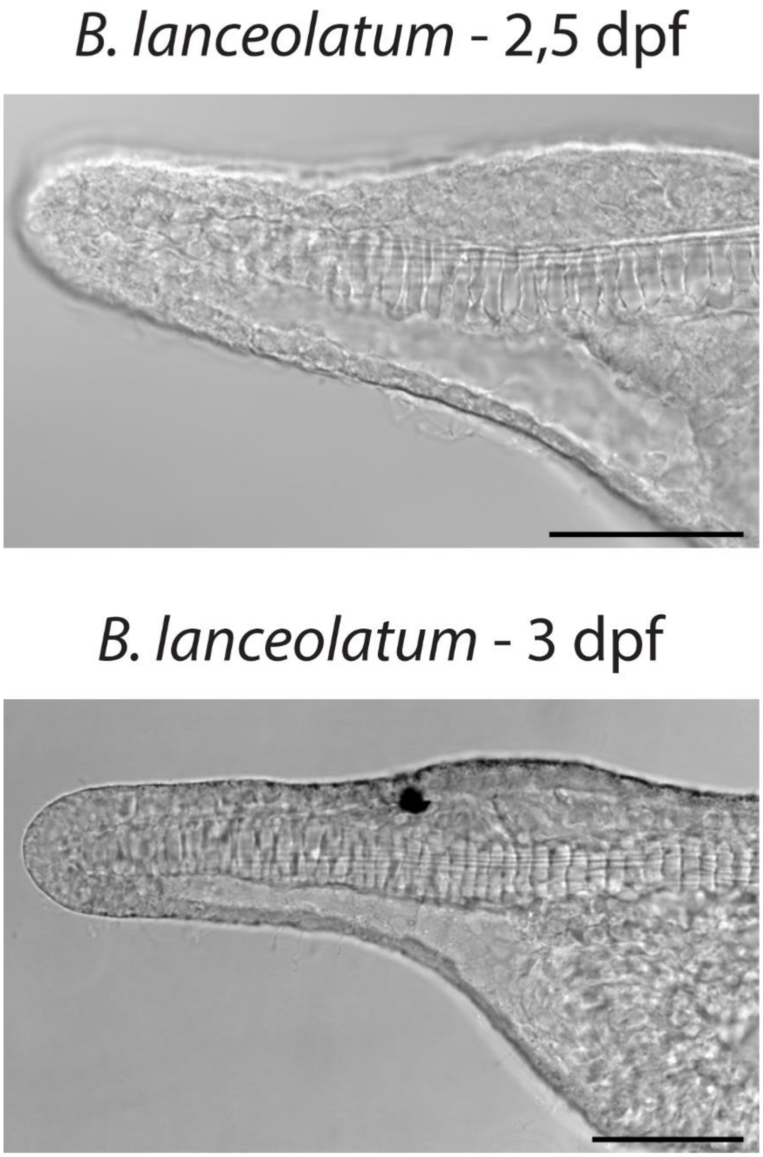

Sup. Fig.1

Pigmentation of *B. lanceolatum* frontal eye

Pigmentation is not visible prior to 3 dpf.

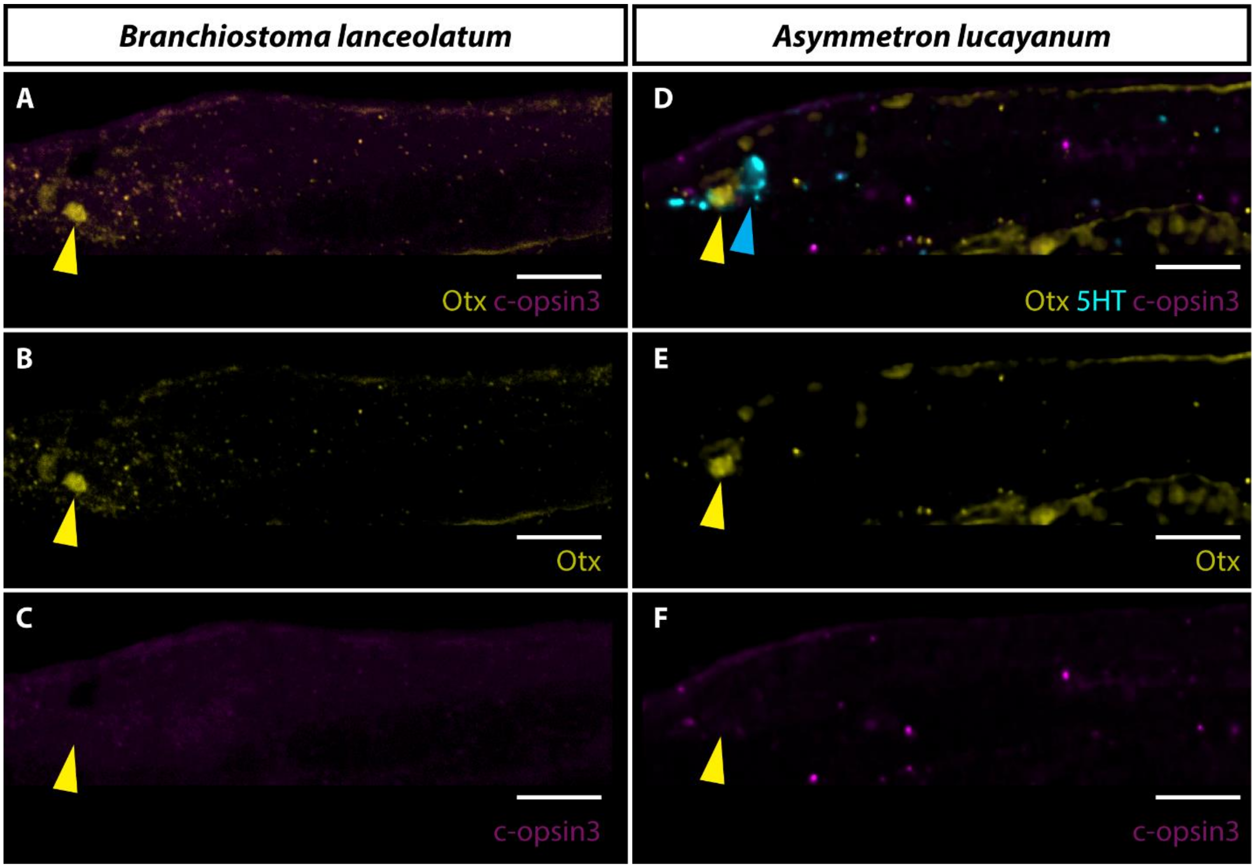

Sup. Fig.2

Staining of *B. lanceolatum* and *A. lucayanum* with c-opsin3 antibody raised against *B. floridae* antigen

A-C – Staining of *B. lanceolatum* 4 dpf old larva with Otx and c-opsin3 antibody raised against *B. floridae* antigen. D-F – Staining of *A. lucayanum* 8 dpf old larva with Otx, 5HT and c-opsin3 antibody. In both cases, it was impossible to obtain signal for c-opsin3 when using *B. floridae* specific antibody. Yellow arrowhead marks Row1 photoreceptors. Cyan arrowhead points to Row2 cells. Scale bar 20 um.

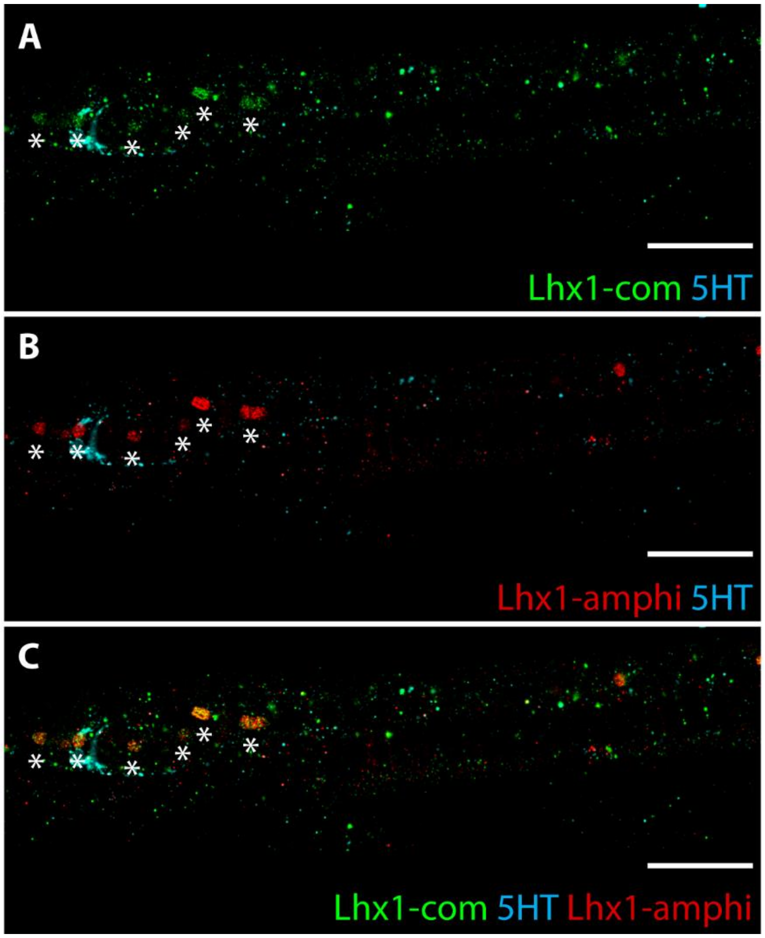

Sup. Fig.3

Comparison of immunostaining using amphioxus-specific Lhx1 antibody and commercially available Lhx1 antibody

A – *B. lanceolatum* 4dpf old larva frontal eye stained with commercially available rabbit antibody against frog Lhx1 (Cat. No. AB3200, Lhx1-com). Asterisks mark Lhx1 positive cells. B – *B. lanceolatum* 4dpf old larva frontal eye stained with amphioxus-specific anti-Lhx1mouse antibody (Lhx1-amphi). C – Overlap of images from A and B. Complete co-localization of both signals was obtained. Scale bar 20 um.

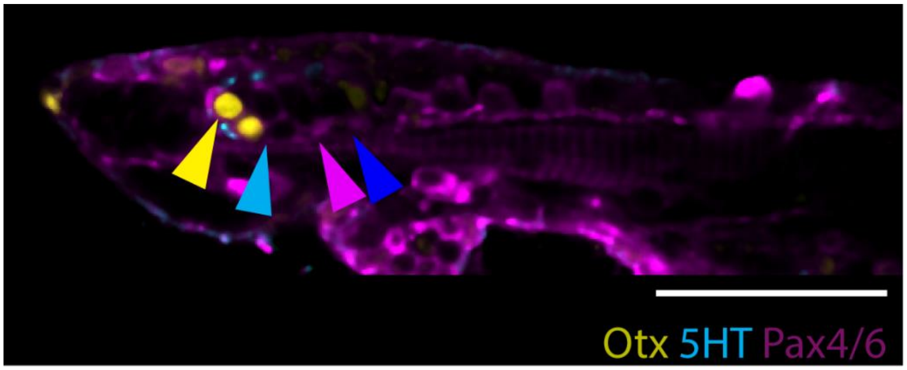

Sup. Fig.4

Expression of glutamate in *A. lucayanum* frontal eye fotoreceptors, Row3 and Row4 neurons

Glutamate is utilized as neurotransmitter in *A. lucayanum* photoreceptors. Row3 and Row4 cells. Yellow arrowhead points to Otx positive Row1 photoreceptors. Pink and blue arrowhead mark Row3 and Row4 neurons positive for glutamate. Scale bar 50 um.

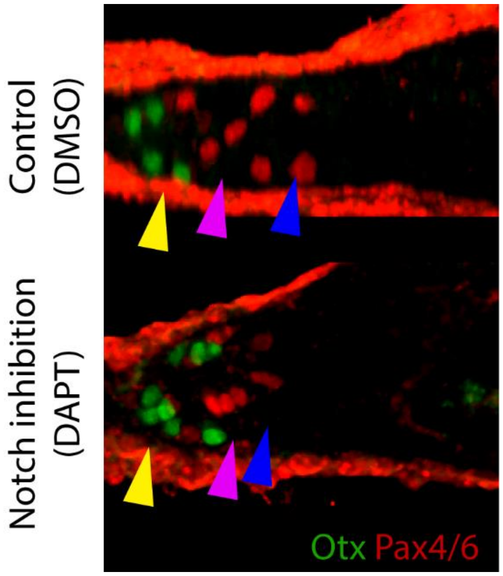

Sup. Fig.5

Comparison of the number of Otx-positive photoreceptors and Pax4/6-positive Row3 and Row4 cells

Inhibition of Notch signaling by DAPT leads to increase of Otx-positive photoreceptors and Pax4/6- positive Row3 and Row4 cells. Due to alteration in cerebral vesicle development after Notch inhibition, are Row3 and Row4 cells less organized when compared with DMSO treated control.

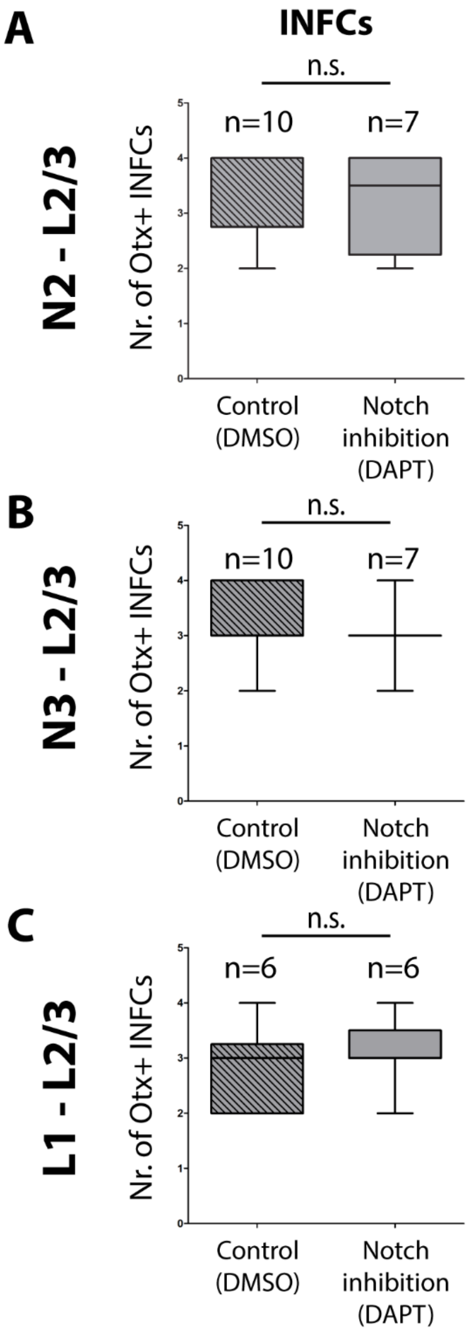

Sup. Fig.6

Result of Notch inhibition on number of infundibular cells (INFCs) in 4dpf larvae of *B. lanceolatum*

Inhibition of Notch signaling by DAPT in any of the periods had no significant effect on number of Otx-positive putative infundibular cells (INFCs).

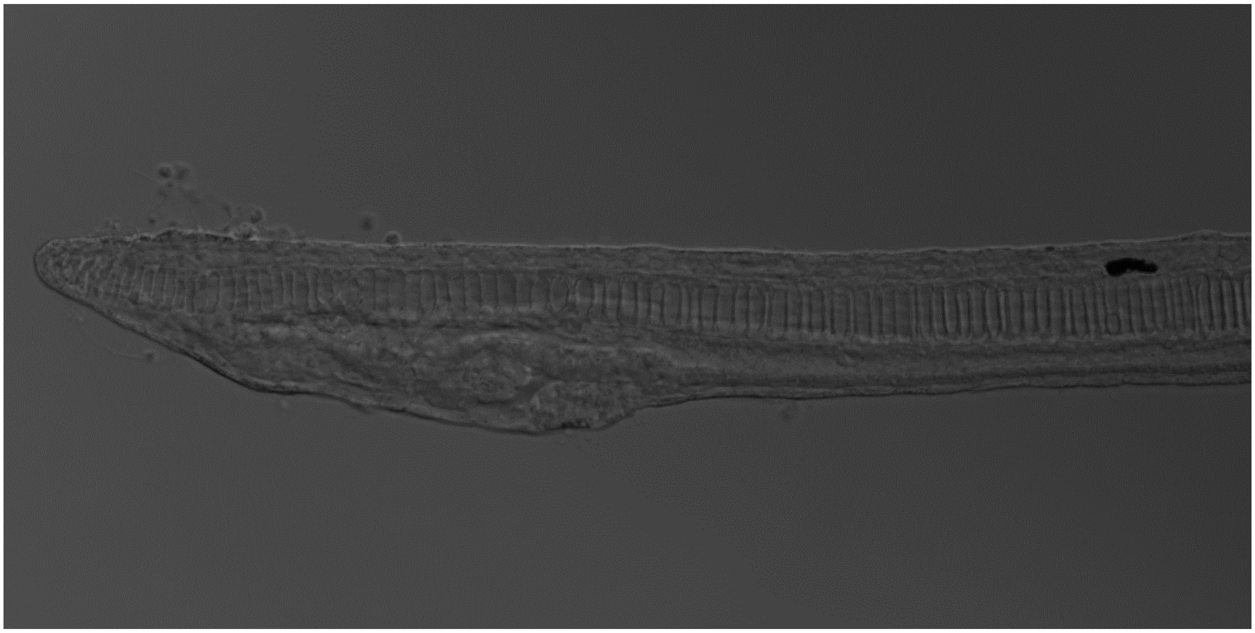

Sup. Fig.7

***A. lucayanum* 8dpf old larva**

The pigmentation of frontal eye is missing, while 1st dorsal ocellus is pigmented.

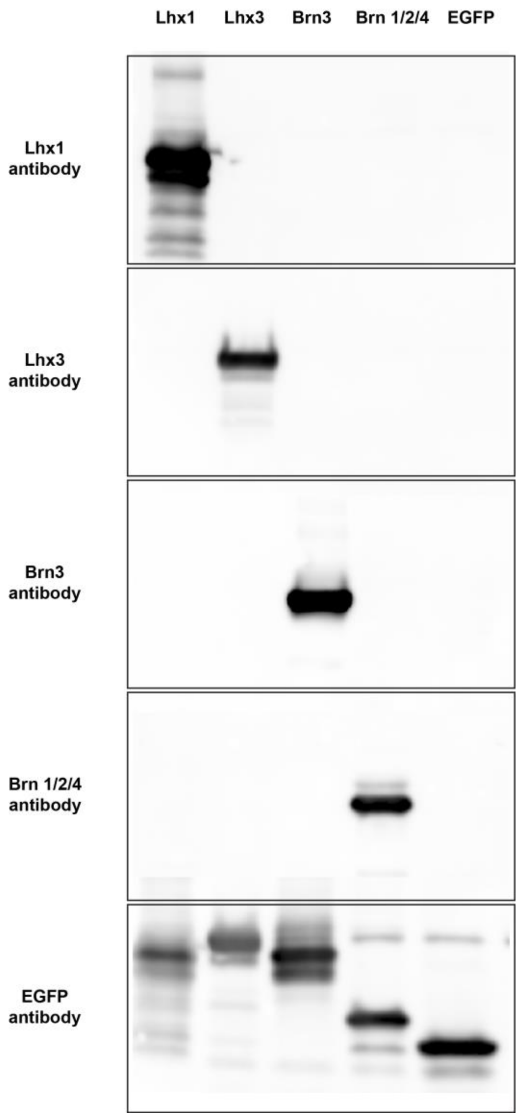

Sup. Fig.8

Western blot validation of Lhx1, Lhx3, Brn3, and Bbrn1/2/4 antibodies.

HEK293T cell lines were transfected with an expression vector carrying coding sequences of the proteins indicated above each lane fused to EGFP or expression vector coding EGFP alone. Two days after transfection a whole-cell extract was prepared and subjected to Western blotting.

To exclude the possible cross-reactivity of antibodies, especially those directed against closely related LIM or POU proteins paralogs, each antibody was tested against all proteins thus showing strict specificity to the respective antigen. In the control Western blot experiment all fusion proteins are recognized by anti-EGFP antibody.

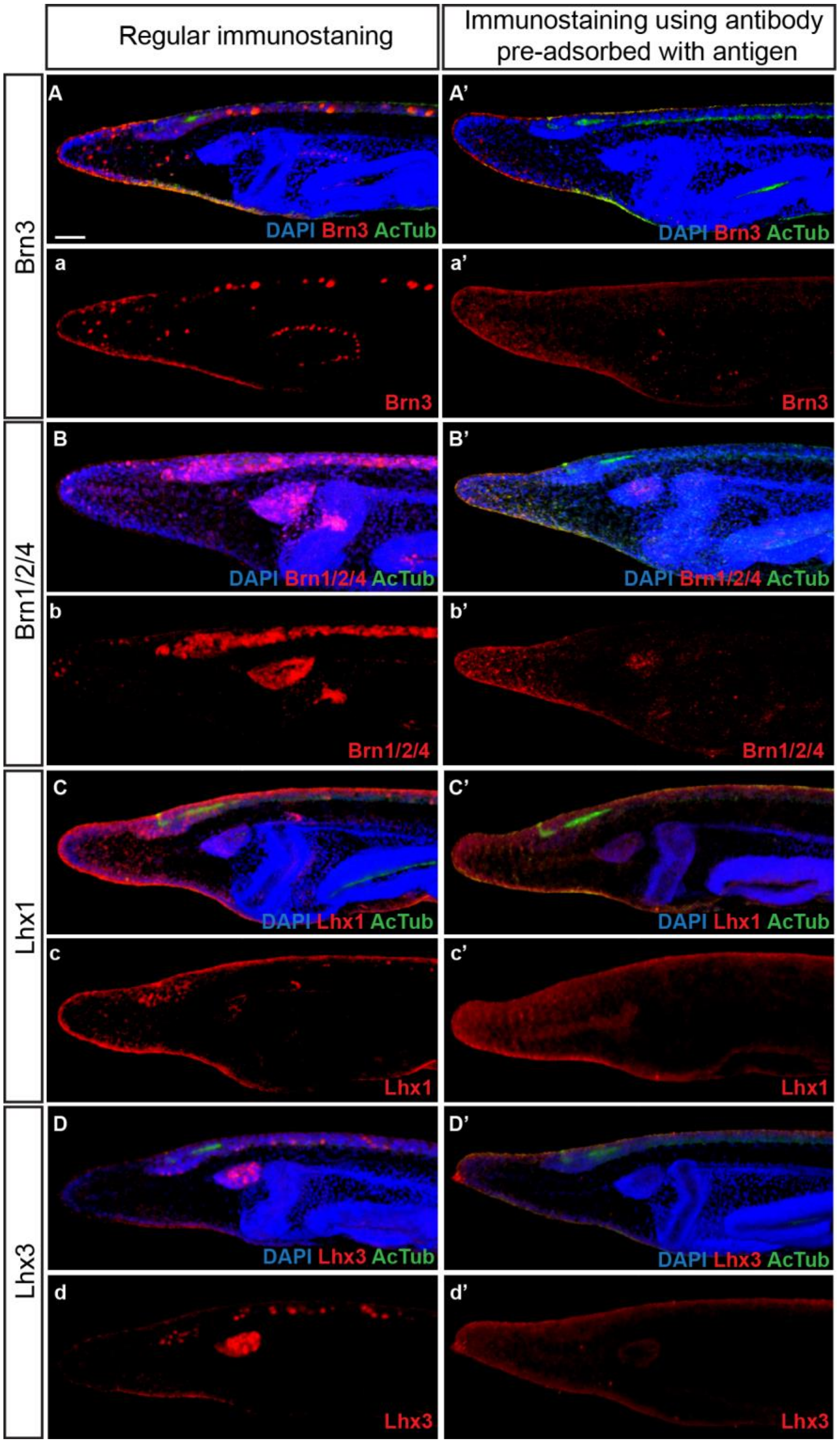

Sup. Fig.9

Validation of Lhx1, Lhx3, Brn3, and Bbrn1/2/4 antibodies by immunostaining.

To validate the specificity of antibodies, we tested for the loss of specific signal after pre-adsorption of the antibody with its respective antigen used for immunization as previously

described (Vopalensky et al., 2012). Purified antigen was blotted to a nitrocellulose membrane, which was subsequently washed three times in phosphate buffered saline and blocked with 10% bovine serum albumin (BSA). Next, the membranes with bound antigen were incubated 6h with antibodies diluted to working concentration in 10% BSA. Following this pre-adsorption, the antibodies were used for immunostaining as described in Methods. As control, the antibody against acetylated tubulin was used to proof specificity of antigen pre-adsorption and to check performance of immunostaining. DAPI was used to label nuclei. Pre-adsorption of Brn3 antibody with Brn3 antigen leads to specific loss of Brn3 staining (compare panels A-a with panels A'-a'). Pre-adsorption of Brn1/2/4 antibody with Brn1/2/4 antigen leads to specific loss of Brn1/2/4 staining (compare panels B-b with panels B'-b'). Pre-adsorption of Lhx1 antibody with Lhx1 antigen leads to specific loss of Lhx1 staining (compare panels C-c with panels C'-c'). Pre-adsorption of Lhx3 antibody with Lhx3 antigen leads to specific loss of Lhx3 staining (compare panels D-d with panels D'-d').
